# Spatial profiling of chronic liver disease: a pilot spatial case series

**DOI:** 10.1038/s41598-026-49400-7

**Published:** 2026-04-20

**Authors:** Shota Sasagawa, Atsushi Ono, Koji Arihiro, Ryoichi Miura, Kazuhiro Maejima, Ayako Ohsawa-Tatsuguchi, Yasuhito Tanaka, Masataka Tsuge, Shiro Oka, Hidewaki Nakagawa

**Affiliations:** 1 Laboratory for Cancer Genomics, RIKEN Center for Integrative Medical Sciences, Yokohama, 230-0045 Japan; 2 Department of Gastroenterology, Graduate School of Biomedical and Health Sciences, Hiroshima University, Hiroshima, 731-8553 Japan; 3 Department of Anatomical Pathology, Hiroshima University Hospital, Hiroshima, 731-8553 Japan; 4 Department of Virology and Liver Unit, Nagoya City University Graduate School of Medical Sciences, Nagoya, 467-8601 Japan; 5 Department of Gastroenterology and Hepatology, Faculty of Life Sciences, Kumamoto University, Kumamoto, 860-8556 Japan; 6 Liver Center, Hiroshima University Hospital, Hiroshima, 731-8553 Japan

**Keywords:** Chronic liver disease, Spatial transcriptomics, MASH, ALD, HCV, Pseudolobule, Computational biology and bioinformatics, Diseases, Gastroenterology, Immunology

## Abstract

The spatial organization of chronic liver disease across major etiologic contexts remains incompletely understood. Here we present a pilot spatial case series using sequencing-based spatial transcriptomics to examine alcohol-associated liver disease (ALD), metabolic dysfunction–associated steatohepatitis (MASH), and chronic hepatitis C within preserved histological context. Eight liver tissues were profiled using a viral-aware workflow enabling in situ detection of hepatitis viral RNA, histology-guided niche annotation, and cell–cell communication analysis; four cases passed quality control and were included in the core discovery analyses. In HCV case, viral reads localized to immune-enriched microenvironments and extended into adjacent regions enriched for cell-cycle, RNA-processing, and PD-1–axis checkpoint pathways. In ALD and MASH tissues, histology-guided segmentation aligned with transcriptomic clusters, and pseudolobular sub-clustering identified recurrent functional compartments. Across the analyzed cases, immune–mesenchymal signaling hubs associated with extracellular matrix and chemokine programs were observed. Overall, this pilot spatial case series suggests that chronic liver disease tissues may share recurrent pseudolobular functional architecture, upon which etiology-associated immune, metabolic, and viral-response programs are superimposed in a case-dependent manner. Supplementary analyses summarize additional feasibility observations from HBV-profiled sections that did not support inclusion in the core downstream analyses.

**Supplementary Information:**

The online version contains supplementary material available at 10.1038/s41598-026-49400-7.

## Introduction

Chronic liver disease is a major global health burden and arises from multiple etiologies, including alcohol-associated liver disease (ALD), metabolic dysfunction–associated steatohepatitis (MASH), and chronic viral hepatitis caused by hepatitis B virus (HBV) and hepatitis C virus (HCV)^1^. Although these diseases share common downstream processes such as chronic inflammation, fibrosis, and hepatocarcinogenesis, they differ in their initiating insults, histopathologic distributions, and tissue microenvironments^1^.

A central challenge in liver pathology is to understand how disease-associated cellular programs are organized within preserved tissue architecture. Bulk RNA-sequencing has identified key molecular pathways involved in chronic liver disease progression, but it averages signals across heterogeneous tissue and obscures spatially restricted biology. Single-cell RNA-sequencing has improved cellular resolution, yet tissue dissociation disrupts the native spatial relationships among hepatocytes, immune cells, and stromal cells^2,3^. Because liver injury progression is shaped by interactions among these cell populations within hepatic lobules and fibrotic septa, approaches that preserve spatial context are needed to understand disease-associated microenvironments^2,4^.

Recent advances in single-cell and spatially resolved transcriptomics have expanded the ability to study liver biology within preserved histological context and to define spatially organized disease programs^2,4^. In hepatocellular carcinoma, spatial studies have identified intratumoral heterogeneity and treatment-associated immune microenvironments^5,6^, whereas in steatohepatitis they have begun to resolve region-specific fibrotic architecture^7^. However, cross-etiology spatial analyses spanning the major chronic liver disease contexts remain limited, and the extent to which recurrent architectural units are preserved or remodeled across ALD, MASH, HBV, and HCV is still incompletely understood.

Here, we performed sequencing-based spatial transcriptomic profiling of eight frozen liver specimens representing ALD, MASH, HBV, and HCV. Because only four cases, one per etiology, passed quality control and were suitable for detailed downstream analysis, we present this work as a pilot spatial case series intended for hypothesis generation rather than patient-level comparative inference. By integrating histopathology, spatial transcriptomics, and a viral-aware mapping workflow, we sought to characterize pseudolobular functional architecture and to examine how etiology-associated immune, metabolic, and viral-response programs are spatially superimposed on preserved liver structure. Our main conclusion is that chronic liver disease tissues may share recurrent pseudolobular functional organization, while disease-associated programs are deployed in a case-dependent spatial manner.

## Results

### Clinical characteristics, quality-control assessment, and study overview

We analyzed eight cases of chronic liver disease (Supplementary Fig. S1), including cases with concomitant hepatocellular carcinoma, with two cases for each etiology: alcoholic liver disease (ALD), metabolic dysfunction–associated steatohepatitis (MASH), chronic hepatitis B (HBV), and chronic hepatitis C (HCV). Fresh-frozen liver resection tissues were processed for Visium spatial transcriptomics. Following quality control (QC) assessment, four cases (R1-ALD-01, R1-MASH-01, R1-HBV-01, R1-HCV-01) met the QC thresholds and were included in the discovery analyses. Because one QC-passed case per etiology was available for the core analyses, cross-etiology observations in this study should be interpreted as case-based spatial comparisons rather than population-level estimates. Supplementary Tables summarizes age, sex, body mass index (BMI), alanine aminotransferase (ALT), fibrosis stage, steatosis and inflammation grades (when applicable), major comorbidities, virologic markers, viral genotype, antiviral treatment history, and Visium QC metrics for each case. Histological evaluation of the ALD and MASH samples was conducted using the Kleiner NAFLD Activity Score (NAS) system, including grading macrovesicular steatosis (Grade 0–3) and necro-inflammatory activity (Grade 0–3). The ALD samples exhibited mild macrovesicular steatosis (~ 20%, Grade 1) with mild inflammation (Grade 1), while the MASH samples showed ~ 5% steatosis (Grade 1) and moderate necro-inflammation (Grade 2). Overall, four of the eight profiled specimens passed the section-level quality-control criteria and were retained for core downstream analysis. The excluded specimens showed insufficient transcript complexity and/or suboptimal section-level quality metrics, underscoring the practical difficulty of applying fresh-frozen spatial transcriptomics to surgically resected fibrotic liver tissues. Accordingly, all downstream findings in this study should be interpreted as exploratory observations derived from a limited pilot case series.

### Optimization of QC and virus detection

**Fig. 1:**
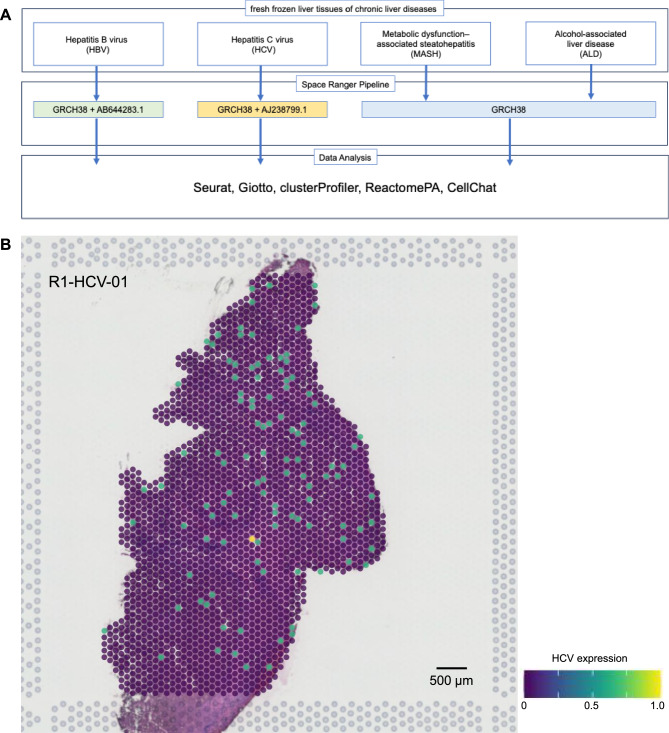
Workflow overview and representative HCV detection in spatial transcriptomics. (**A**) Study workflow using a composite hg38 + HBV + HCV reference and downstream Seurat/CellChat analyses. (**B**) Representative spatial feature plot of HCV-derived reads in the QC-passed HCV discovery case (R1-HCV-01). Data-derived plots were generated by the authors using R version 4.4.3 (https://www.r-project.org/) together with relevant software packages according to the plot type.

Workflow of the Visium data analysis and viral-read detection is shown in Fig. 1A. To enhance the sensitivity for detecting virus-derived reads in spatial transcriptomics data, we constructed a custom reference genome by integrating the full HBV genome (GenBank: AB644283.1) and the full HCV genome (GenBank: AJ238799.1) into the standard human reference genome (hg38). This enabled simultaneous mapping of human gene expression and viral sequences with high sensitivity. Across the four QC-passed samples, mean total reads per spot ranged from approximately 2.1–2.8 × 10^5 (mean = 247,898), and median genes per spot ranged from ~ 1000–1800 (mean = 1,323), indicating robust data quality suitable for downstream analyses. In the HCV case (R1-HCV-01), HCV-derived reads were readily detected in patchy areas of the liver tissue (Fig. 1B), with corresponding viral-read coverage shown in Supplementary Fig. S2A.

We extended this analysis to the second cohort (R2-HBV-01 and R2-HCV-01) (Supplementary Fig. S2B, S2C). Spatial clustering using Seurat’s SpatialDimPlot revealed broadly comparable section-level spatial organization across the first (R1) and second (R2) cohorts, with preserved cluster structures despite differences in viral-read detectability. In R2-HCV-01, no HCV-derived reads were detected in the analyzed section (Supplementary Fig. S2C), consistent with the corresponding serum HCV viral load being below the detection limit (Supplementary Fig. S2D). These observations indicate that section-level spatial clustering patterns were qualitatively reproducible across the available cohorts, whereas viral-read detectability varied across specimens according to virologic status, section selection, and assay sensitivity.

HBV-profiled sections were also evaluated in the viral-aware workflow, and the corresponding tissue-level observations are summarized in Supplementary Fig. S2B. Specifically, R1-HBV-01 passed section-level QC but showed no detectable intrahepatic HBV-derived reads in the analyzed section, precluding the definition of HBV-positive spatial regions. In contrast, R2-HBV-01 showed sparse HBV-derived signal, but this section did not pass the QC thresholds required for robust downstream analysis. For clarity, these HBV-related observations are summarized in Supplementary Table and Supplementary Figure S2 rather than being carried forward into the main comparative analyses.

Serum viral load measured by qPCR was 6.3 log IU/mL for R1-HCV-01 and below the detection limit for R1-HBV-01. In the second cohort, serum HCV viral load in R2-HCV-01 was below the detection limit, whereas R2-HBV-01 had detectable HBV viremia (2.4 log IU/mL) (Supplementary Fig. S2D). Because viral-read detection in individual sections may be influenced by section selection, pre-analytic variables, and platform sensitivity, the absence of detectable viral reads in a given section should not be interpreted as evidence against the underlying clinical diagnosis, nor should preservation of clustering structure be interpreted as evidence of preserved intrahepatic viral localization.

### Spatial distribution of steatosis- and inflammation-associated niches across ALD, MASH, and HCV

**Fig. 2:**
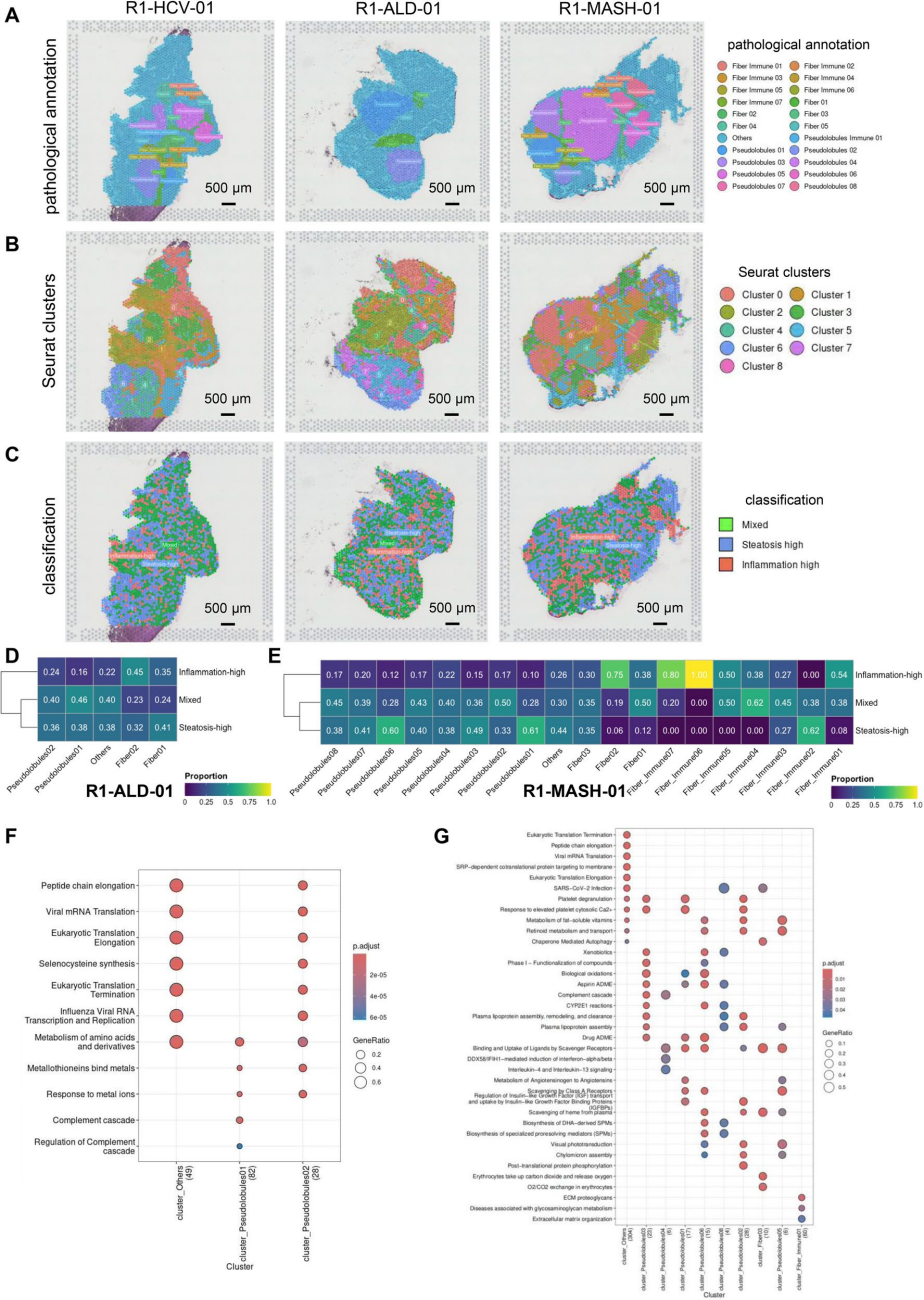
Integrative analysis of spatial transcriptomics with pathological annotations across ALD, MASH, and HCV. (**A**) Manually defined anatomical regions in the ALD, MASH, and HCV discovery cases based on histopathological features. (**B**) Spatial distribution of Seurat-derived clusters in the ALD, MASH, and HCV discovery cases, demonstrating spatial correspondence with the manual annotations. (**C**) Spatial mapping of spots classified into three groups—Steatosis-high, Inflammation-high, and Mixed—by k-means clustering (k = 3) using steatosis and inflammation module scores. (**D**, **E**) Detailed subregion-level heatmaps for the ALD and MASH cases. (**F**, **G**) Bubble plots of GO and Reactome pathway enrichment for the top differentially expressed genes in ALD and MASH regions. Data-derived plots were generated by the authors using R version 4.4.3 (https://www.r-project.org/) together with relevant software packages according to the plot type.

Histology-guided segmentation of Visium sections first defined the major pathological compartments in the ALD, MASH, and HCV discovery cases (Fig. 2A). These manually annotated regions showed close spatial correspondence with the Seurat-derived clusters in each case, indicating that unsupervised transcriptomic structure broadly recapitulated the underlying histo-anatomical organization (Fig. 2B**)**. We then applied steatosis and inflammation module scores followed by k-means clustering (k = 3) to classify capture spots into Steatosis-high, Inflammation-high, or Mixed groups (Fig. 2C).

To facilitate cross-context comparison despite differences in the number of manually annotated subregions, we additionally summarized the module-defined spatial classes at the level of broad histo-anatomical compartments. At this broader level, ALD and MASH retained compartment-biased distributions of Steatosis-high and Inflammation-high spots, whereas the HCV case showed a more heterogeneous composition across compartments (Supplementary Fig. S3). We then returned to the finer subregion-level annotations for the ALD and MASH cases, in which the steatosis/inflammation framework was originally developed and could be more directly linked to region-specific pathway analysis. In ALD, steatosis-high spots were enriched in pseudolobular compartments, whereas inflammation-high spots were enriched in immune-cell–rich fibrotic septa (Fig. 2D). In MASH, immune-cell–rich fibrotic regions were predominantly inflammation-high, whereas several pseudolobular regions showed enrichment of Mixed spots, suggesting co-existence of steatosis and inflammation (Fig. 2E). Region-specific differential expression analysis revealed enrichment of complement cascade regulation and metabolic programs in ALD (Fig. 2F), versus cytokine signaling and extracellular matrix remodeling in MASH (Fig. 2G), indicating distinct pathological drivers in each steatotic disease context. In the HCV case, the module-based classification showed a more heterogeneous spatial composition and is presented here for qualitative cross-context comparison; HCV-specific spatial organization was examined separately using the viral-proximity framework in Fig. 4.

### Pseudolobular functional architecture across diseases

**Fig. 3:**
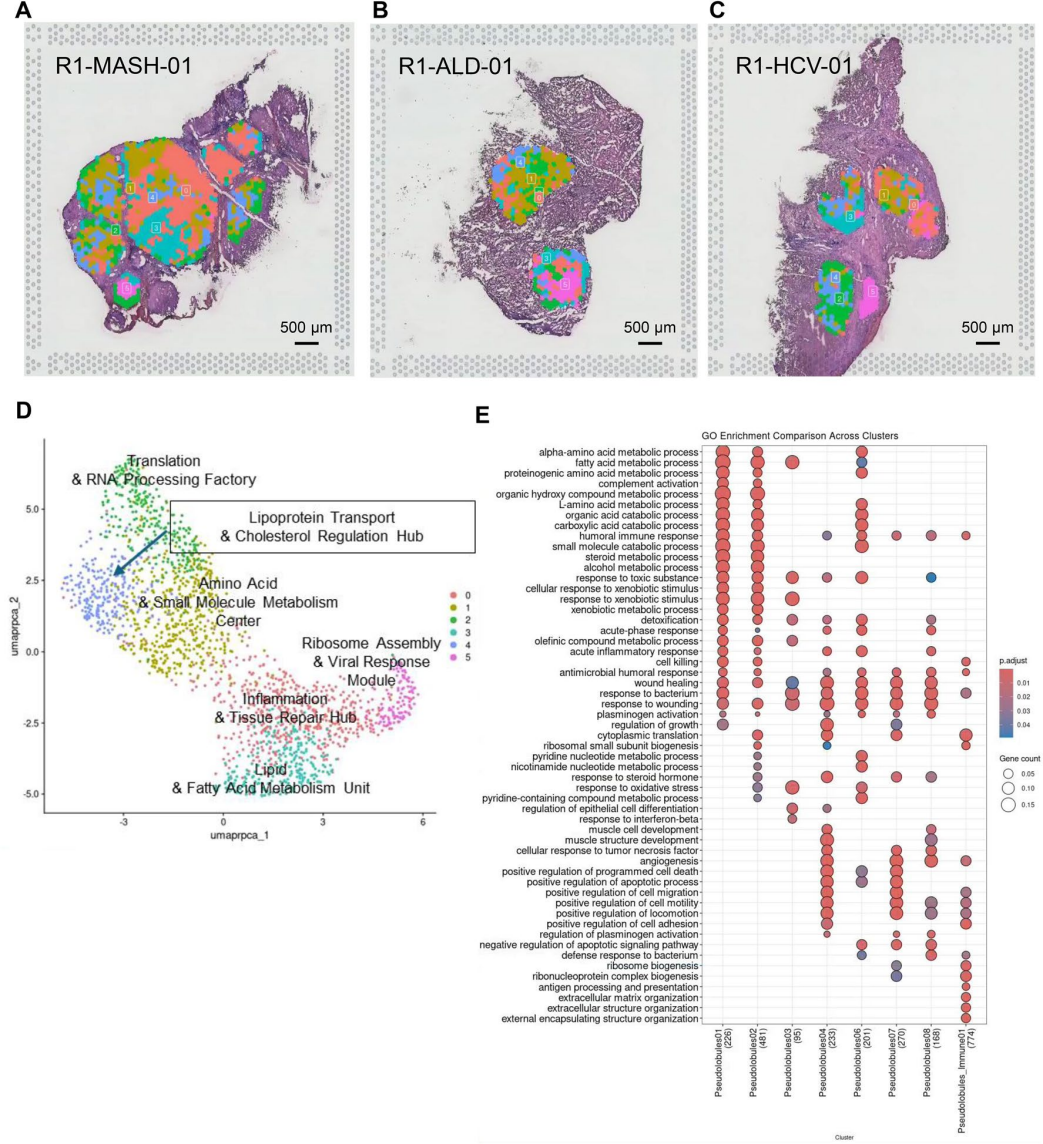
Pseudolobular sub-clustering and functional compartments. (**A**–**C**) FeatureSpatialPlots of pseudolobular sub-clusters across MASH, ALD, and HCV. (**D**) UMAP of pooled pseudolobular spots showing frequent co-clustering of spots within individual pseudolobules. (**E**) GO enrichment highlighting six functional compartments. Data-derived plots were generated by the authors using R version 4.4.3 (https://www.r-project.org/) together with relevant software packages according to the plot type.

A key finding emerged from the sub-clustering of all pseudolobular regions combining MASH, ALD, and HCV datasets (Fig. 3A–C). We observed that spots originating from the same physically defined pseudolobule consistently grouped together in the UMAP embedding (Fig. 3D), indicating that each pseudolobule represents a distinct, transcriptionally coherent unit, potentially corresponding to recurrent functional domains within the hepatic architecture. This analysis resolved the pseudolobules into at least six distinct functional compartments whose enrichment profiles were defined by gene ontology signatures (Fig. 3D, E): an Inflammation & Tissue Repair Hub (Cluster 0), characterized by wound healing, angiogenesis, cell chemotaxis, and fibroblast growth factor responses; an Amino Acid & Small Molecule Metabolism Center (Cluster 1), enriched for amino acid catabolic and biosynthetic processes and organic acid metabolism; a Translation & RNA Processing Factory (Cluster 2), dominated by ribosome biogenesis, mRNA splicing, and translational regulation; a Lipid & Fatty Acid Metabolism Unit (Cluster 3), marked by fatty acid metabolism, lipid remodeling, and organic hydroxy compound metabolism; a Lipoprotein Transport & Cholesterol Regulation Hub (Cluster 4), containing high-density lipoprotein particle clearance, phospholipid efflux, and negative regulation of cholesterol transport; and a Ribosome Assembly & Viral Response Module (Cluster 5), enriched for ribosome biogenesis and viral process terms (Fig. 3E). Together, these findings reveal that pseudolobules harbor reproducible and spatially organized functional specializations, which are preserved across disease contexts yet exhibit disease-specific prevalence patterns, reflecting both conserved hepatic architecture and dynamic remodeling in chronic liver disease.

### Spatial localization of HCV and host response

**Fig. 4:**
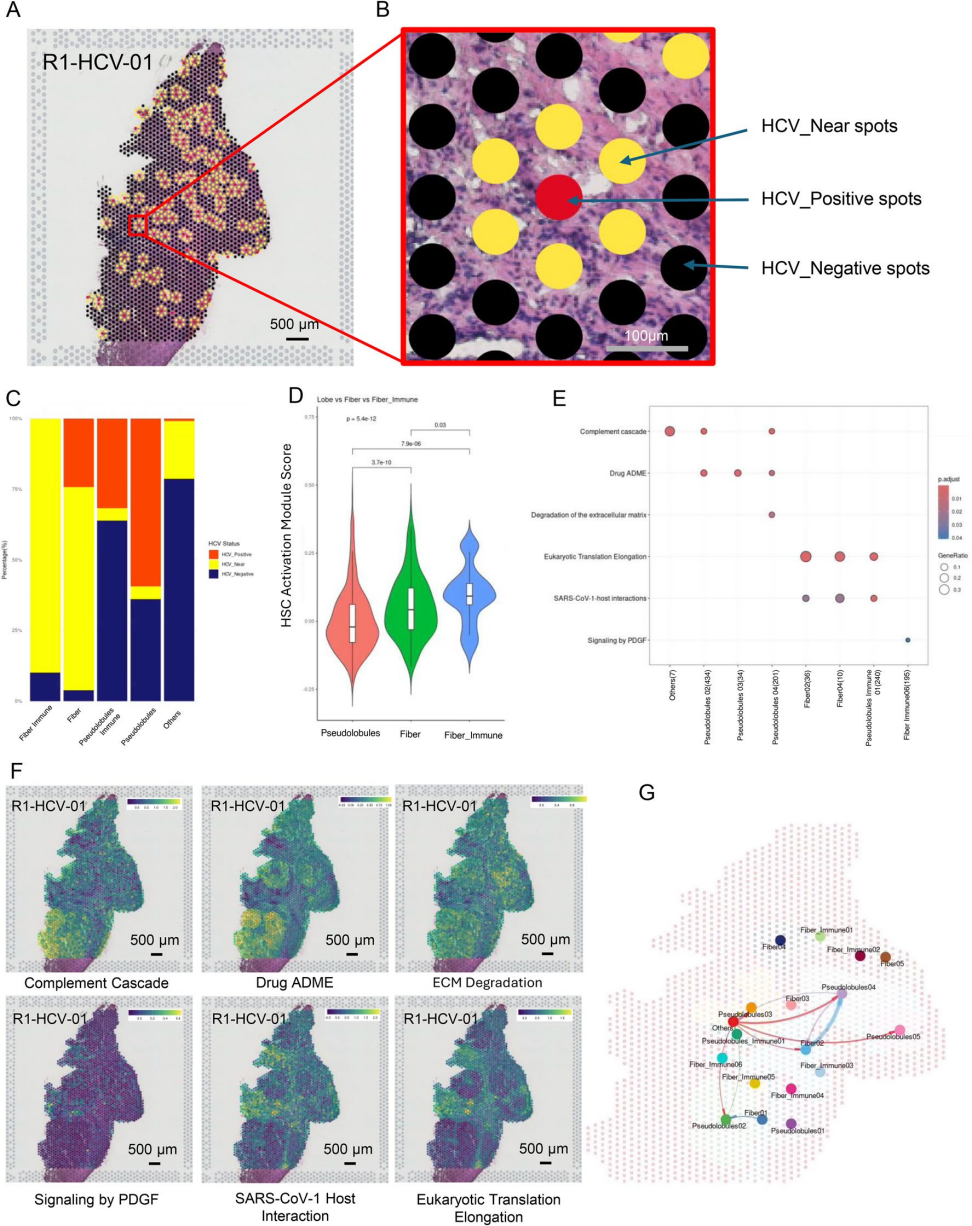
HCV-centered spatial analysis of viral localization and host response. (**A**) Spatial localization of HCV-derived reads across the HCV discovery section. Pathological interpretation was guided by the annotation framework shown in Fig. 2A. (**B**) Classification of Visium spots into HCV_Positive, HCV_Near, and HCV_Negative groups according to detectable HCV-derived reads and spatial proximity to viral signal. (**C**) Proportions of spot clusters across HCV_Positive, HCV_Near, and HCV_Negative regions. (**D**) HSC activation scores across major anatomical compartments. (**E**) Bubble plot of representative pathways enriched in distinct spatial clusters. (**F**) Heatmap of pathway activity scores for complement cascade, drug ADME, ECM degradation, PDGF signaling, SARS-CoV-1 host interaction, and translation elongation across spatial clusters. (**G**) CellChat-based ligand–receptor interaction network inferred among spatial spot clusters. Data-derived plots were generated by the authors using R version 4.4.3 (https://www.r-project.org/) together with relevant software packages according to the plot type.

To complement the shared histology-guided comparison shown in Fig. 2, we next analyzed the HCV case using an HCV-centered spatial framework. HCV-derived reads were mapped across the Visium section using our optimized detection pipeline, revealing discrete viral-signal microenvironments within the liver parenchyma (Fig. 4A). Spatial interpretation was guided by the pathological annotations shown in Fig. 2A, which subdivided the tissue into Fiber, Fiber_Immune, Pseudolobules, and Pseudolobules_Immune compartments. HCV-derived reads were enriched in the immune-associated Fiber_Immune and Pseudolobules_Immune compartments, detected at intermediate levels in Pseudolobules, and least abundant in acellular Fiber septa, indicating preferential localization of viral signal to immune-rich niches.

Each Visium spot was then classified as an HCV_Positive spot, HCV_Near spot, or HCV_Negative spot according to detectable HCV-derived reads and spatial proximity to viral signal (Fig. 4B), enabling direct comparison of transcriptional states across viral-signal–associated and distal regions. In HCV_Positive areas, Fiber_Immune components were largely absent, whereas mild recruitment was observed in HCV_Near regions and highest in HCV_Negative regions, suggesting a spatial immune gradient (Fig. 4C, and Supplementary Fig. S4A). Pseudolobules were most prevalent near HCV-read-positive areas, decreasing toward distal regions, while the proportion of Fibers remained constant and Others consistently accounted for > 60%.

Gene expression analysis suggested a possible upregulation of immune checkpoint molecules (*PDCD1, CTLA4, LAG3, HAVCR2, TIGIT*) in HCV_Positive regions, whereas macrophage-associated markers, interferon-stimulated genes (ISGs), T-cell signature genes, and chemokines showed little apparent difference (Supplementary Fig. S4B–F). To further assess whether HCV-derived reads in immune-rich regions were more consistent with hepatocyte-associated localization or immune-cell-associated uptake of viral material, we examined the relationship between HCV-derived read abundance and expression of hepatocyte markers (*ALB, HNF4A, APOB, TTR*) and macrophage markers (*CD68, LYZ, MARCO, CD163*) (Supplementary Fig. S4G, H). Across all spots, HCV-derived read abundance showed weak positive correlations with several hepatocyte-associated genes, whereas canonical macrophage markers did not show positive correlation (Supplementary Fig. S4G). Consistent with this trend, Loupe-defined HCV-associated regions retained expression of hepatocyte markers without broad enrichment of canonical macrophage markers (Supplementary Fig. S4H). However, because Visium spots capture mixed-cell transcripts, these data do not by themselves distinguish productive infection of hepatocytes from uptake or processing of viral material. Hepatic stellate cell (HSC) activation scores (Fig. 4D) tended to be higher in Fiber_Immune compared with Pseudolobules, which may indicate localized induction of fibrogenic signaling pathways (e.g., TGF-β, PDGF).

Pathway analysis of 512 spots proximal to HCV_Positive regions revealed enrichment in mitotic control (e.g., mitotic anaphase), RHO GTPase signaling (RHOG, RAC2), mRNA splicing, and host pathways involved in SARS-CoV interactions (Supplementary Fig. S5). Pathway enrichment in distinct spatial clusters (Fig. 4E, and Supplementary Fig. S6) showed that Pseudolobules clusters (Pseudolobules_02, _03, _04) were enriched for complement cascade, drug ADME, plasma lipoprotein assembly/remodeling/clearance, and fibrin clot formation, whereas degradation of the extracellular matrix was specific to Pseudolobules_04 and _05. Fiber clusters (Fiber_02, Fiber_04) were dominated by protein synthesis pathways, most notably eukaryotic translation elongation. Immune clusters demonstrated the strongest enrichment in translational machinery, viral response pathways, and immune signaling, including antigen presentation and PD-1 signaling. Spatial distributions of these pathway activities are illustrated in Fig. 4F. CellChat-based analysis identified ligand–receptor co-expression patterns, particularly involving ANGPTL, SPP1, and IGFBP families, concentrated in Fiber_02 and Pseudolobules_04 clusters (Fig. 4G). Because Visium spots contain mixed-cell transcripts and no deconvolution was performed, these results should be interpreted as exploratory spot-level signaling patterns. These findings suggest spatially structured intercellular communication in HCV-associated tissue microenvironments. Collectively, these results highlight a finely tuned host–virus interplay involving cell-cycle regulation, RNA processing, metabolism, and extracellular matrix remodeling, and point to pathways such as mRNA splicing, RHO GTPase activity, and CYP450 metabolism as candidate pathways for further evaluation.

### Exploratory cross-case comparison and candidate pathways

**Fig. 5:**
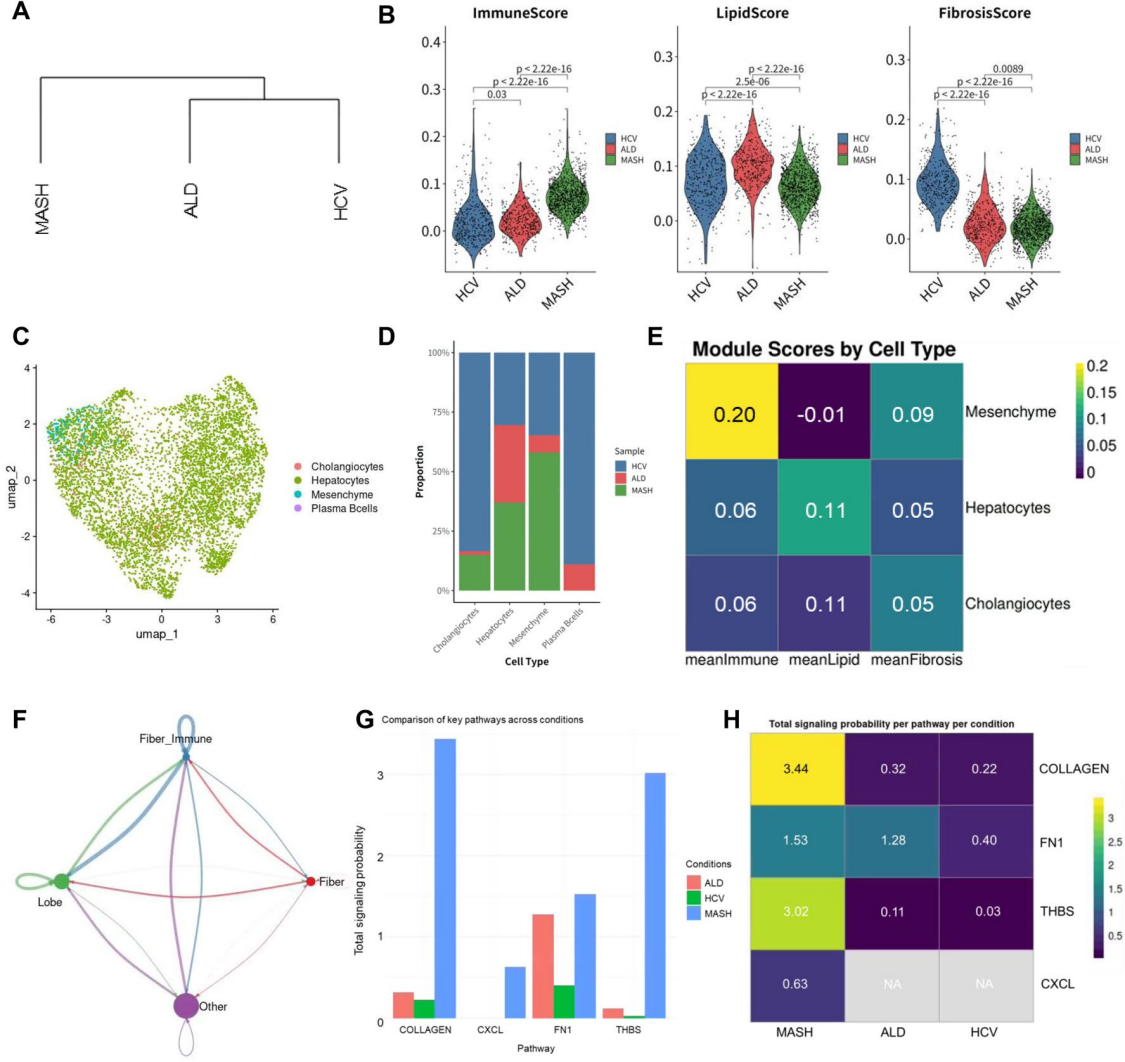
Comparative analysis of disease-specific gene expression, cellular composition, and signaling networks across ALD, MASH, and HCV models. (**A**) Hierarchical clustering of gene expression profiles from ALD, MASH, and HCV models, showing that ALD and HCV group closely, while MASH forms a separate but related cluster. (**B**) Violin plots of module scores for immune response, lipid metabolism, and fibrosis across disease models. MASH has the highest immune response score, ALD the highest lipid metabolism score, and HCV the highest fibrosis score. (**C**) UMAP plot visualizing identified cell clusters, including hepatocytes, cholangiocytes, mesenchymal cells, and plasma B cells. (**D**) Sample composition within each cell type (cholangiocytes, hepatocytes, mesenchymal cells, and plasma B cells). (**E**) Module scores for immune response, fibrosis, and lipid metabolism in major cell types. Mesenchymal cells exhibit the highest scores for immune response and fibrosis, whereas hepatocytes are dominant in lipid metabolism. (**F**) Cell–cell communication network analysis by CellChat, demonstrating that Fiber_Immune regions function as signaling hubs, interacting with Lobe regions via ECM (COLLAGEN, THBS) and chemokine (CXCL) pathways. (**G**) Circle plot illustrating the activity of major signaling pathways in Lobe and Fiber_Immune regions. (**H**) Comparison of COLLAGEN, THBS, and CXCL signaling pathway activity across each disease model, highlighting the prominent activation of these pathways in MASH. Data-derived plots were generated by the authors using R version 4.4.3 (https://www.r-project.org/) together with relevant software packages according to the plot type.

Exploratory hierarchical clustering was restricted to the ALD, MASH, and HCV discovery cases because the QC-passed HBV section (R1-HBV-01) lacked spatially detectable intrahepatic HBV reads, while the additional HBV section with sparse HBV-derived reads (R2-HBV-01) did not pass QC for robust downstream analysis (Fig. 5A). By calculating module scores for feature expression programs in single spots, we highlighted disease-specific drivers (Fig. 5B): immune activation was highest in MASH (~ 0.04), lipid metabolism in ALD (~ 0.13), and fibrosis in HCV (~ 0.11). Cell-type annotation (Fig. 5C–E) showed mesenchymal cells with the highest immune (~ 0.20) and fibrosis (~ 0.09) scores, and hepatocytes dominating lipid metabolism (~ 0.11). Cell–cell communication network analysis by CellChat positioned Fiber_Immune regions as signaling hubs via ECM (COLLAGEN, THBS) and chemokine (CXCL) pathways, interacting bidirectionally with pseudolobules (Fig. 5F–H). MASH displayed prominent ECM and chemokine activation, highlighting antifibrotic and chemokine-related pathways as candidate axes for future investigations.

## Discussion

In this pilot spatial case series, we used spatial transcriptomics to examine whether recurrent spatial organization could be observed across a small number of chronic liver disease specimens with different clinical backgrounds. Rather than defining etiology-specific tissue programs, our analyses suggest that pseudolobular units may provide a useful spatial framework onto which immune, metabolic, fibrotic, and viral-response programs are superimposed in a case-dependent manner. Within the analyzed specimens, these observations offer an exploratory map of spatial heterogeneity and generate hypotheses for future validation in larger and clinically better-matched cohorts.

In HCV, capture spots nearer to HCV-read-positive areas showed higher expression of immune-checkpoint molecules with only modest changes in canonical interferon-stimulated genes and macrophage/T-cell signatures, indicating a localized equilibrium between viral immune evasion and host elimination. Enrichment of mitotic control, RHO GTPase signaling, and RNA processing around HCV-associated niches accords with CD81-dependent entry and Rac1/Cdc42-Raf/MEK/ERK activation^8^, suggesting that post-entry host pathways may contribute both to viral persistence and to a tumor-permissive microenvironment. Together with prior evidence that chronic inflammation, ROS, and NF-κB/TNF-α signaling promote proliferation with apoptosis resistance and angiogenesis^9^, these spatial observations highlight the checkpoint axis, RNA-processing and RHO-associated signaling as candidate pathways for further evaluation HCV-related disease.

Across the analyzed cases, mesenchymal populations showed the strongest immune- and fibrosis- associated programs, whereas hepatocytes were enriched for lipid metabolic functions. Network analysis positioned immune-enriched fibrotic septa as hubs transmitting ECM (COLLAGEN/THBS) and chemokine (CXCL) cues into the parenchyma, with these features appearing particulary prominent in the analyzed MASH case. This pattern is compatible with the multiple-hit model and lipotoxicity concept^10^, in which cytotoxic lipids are expected to activate immune and profibrotic signaling. In this context we observed immune-cell–rich septa linked to ECM remodeling—overlying parenchyma enriched for complement, drug ADME/CYP450 metabolism, and lipoprotein assembly/remodeling. These spatial couplings suggest a mechanistic link between lipid-associated stress and immune-fibrotic activation in MASH.

In ALD, steatosis-high pseudolobules with metabolic program shifts and parenchymal complement/ADME enrichment are consistent with increased FFA influx, AMPK inhibition with SREBP-1c induction, reduced β-oxidation via PPARα downregulation, and ethanol-mediated suppression of gluconeogenesis^11^. The spatial organization also aligns with recognized histopathologic features, including portal-tract-predominant fibrosis and sclerosing hyaline necrosis^12^, and the reduced visibility of lipid droplets in advanced fibrosis is compatible with late-stage metabolic liver injury. Collectively, these observations suggest that metabolic perturbation is a major component of the ALD-associated niches identified here, whereas immune/ECM circuitry appeared more prominent in the analyzed MASH case and fibrosis-linked immune programs characterized the analyzed HCV microenvironment.

One notable observation in the present dataset was the partitioning of pseudolobules into six functional compartments—Inflammation/Tissue Repair; Amino Acid/Small Molecule Metabolism; Translation/RNA Processing; Lipid/Fatty Acid Metabolism; Lipoprotein Transport/Cholesterol Regulation; and Ribosome Assembly/Viral Response—with spots from the same anatomical pseudolobule often co-clustering. This spatial organization may inform future sampling strategies, pathology readouts, and candidate spatial biomarker development by indicating that the pseudolobular unit could be a biologically relevant level of analysis. The ligand–receptor landscape further refines potential disease-associated signaling axes. Concentrated ANGPTL, SPP1, and IGFBP interactions within specific fibrotic and pseudolobular clusters indicate spatially structured intercellular communication. In the analyzed MASH case, where ECM and chemokine networks were particularly prominent, these pathways may represent candidate signaling axes for future mechanistic investigation.

In the analyzed ALD case, metabolic interventions targeting the SREBP-1c/PPARα axis, together with consideration of ADME/CYP heterogeneity in steatotic parenchyma, may warrant further study. In the analyzed HCV case, modulation of the immune microenvironment, alongside exploratory evaluation of RNA-processing or RHO GTPase–linked pathways, may help generate future mechanistic and translational hypotheses, while acknowledging the pleiotropic biology of these pathways.

In summary, this pilot spatial case series suggests that conserved pseudolobular spatial compartments may be engaged differently across the analyzed ALD, MASH, and HCV cases. The resulting map provides a preliminary framework linking canonical disease-associated mechanisms to discrete tissue niches. These observations contextualize spatial heterogeneity within the analyzed specimens and generate hypotheses for future validation, rather than supporting definitive etiology-level or translational conclusions. Given the overlapping metabolic and alcohol-related backgrounds present across cases, the observed spatial patterns should not be interpreted as exclusive signatures of any single etiology.

### Limitations

This study has several important limitations. First, although eight resection specimens were profiled, only four QC-passed cases were included in the core discovery analyses, leaving one analyzed case per etiology. Accordingly, this work should be interpreted as a pilot spatial case series rather than a comparative population study. Second, substantial inter-patient heterogeneity likely influenced the observed spatial patterns, including differences in fibrosis stage, background liver condition, overlapping alcohol-related and/or metabolic comorbidities, treatment exposure, virologic status, and detectability of intrahepatic viral RNA. In particular, although R1-HBV-01 was clinically classified as an HBV case, no intrahepatic HBV reads were detected in the analyzed section, so this specimen could not support direct spatial comparison of viral localization with the analyzed HCV case. Third, the analyses focused on resection tissues, including cases with advanced fibrosis (F3–4) and tumor-related clinical contexts, which may not fully represent chronic liver disease more broadly. Fourth, statistical comparisons were performed at the level of capture spots or histology-defined regions within tissue sections and therefore do not constitute patient-level inference across etiologies. Fifth, we did not perform orthogonal viral-protein validation, such as HBsAg or HCV core antigen immunofluorescence on consecutive sections, for the analyzed specimens. Therefore, the spatial viral signals reported here should be interpreted as transcript-derived observations that warrant further validation by complementary imaging-based assays. Finally, platform sensitivity for low-abundance transcripts and pre-analytic factors may have contributed to negative viral detection in some specimens. Future studies should expand cohorts across etiologies and earlier fibrosis stages, include clinically more homogeneous non-tumor tissues, and integrate longitudinal spatial profiling with orthogonal approaches such as spatial multi-omics, lineage analysis, or mutation-based methods to test hypotheses regarding pseudolobular organization and potential clonality.

## Methods

### Ethics and sample acquisition

Resected liver tissues from patients with alcohol-associated liver disease (ALD), metabolic dysfunction–associated steatohepatitis (MASH; clinically diagnosed as non-alcoholic steatohepatitis, NASH), HBV infection, or HCV infection in the setting of hepatocellular carcinoma (HCC) were obtained at surgery. Each case was selected according to established clinical and pathological diagnostic criteria, and expert hepatologists and pathologists reviewed all diagnoses. Written informed consent was obtained from all patients prior to tissue collection. The study protocol was approved by the institutional review boards at RIKEN and Hiroshima University Hospital (approval no. E2012-9998), and all procedures complied with relevant ethical regulations for research involving human participants.

### Histopathology

Formalin-fixed, paraffin-embedded (FFPE) liver specimens were sectioned and stained with hematoxylin and eosin (H&E) for histopathological assessment. Experienced hepatopathologists evaluated fibrosis stage, steatosis, and necroinflammatory activity. For steatohepatitis cases, the NAFLD Activity Score (NAS) system was applied to grade macrovesicular steatosis and necroinflammation, facilitating comparison with historical non-alcoholic fatty liver disease (NAFLD)/NASH datasets and mapping to MASH activity as described in the main text.

### Visium spatial transcriptomics (fresh-frozen) and viral RNA detection

Fresh liver resection specimens were rapidly embedded and cryosectioned at 10 µm thickness. Sections were processed using the Visium Spatial Gene Expression Fresh-Frozen workflow (10 × Genomics) according to the manufacturer’s instructions. After tissue permeabilization and reverse transcription on the capture areas, cDNA libraries were constructed and sequenced on an Illumina NovaSeq platform.

Quality-control (QC) assessment was performed at both the spot and section levels. Spot-level QC considered the total number of sequence reads per spot and the number of genes detected per spot. Section suitability for downstream analysis was assessed using Space Ranger web_summary.html outputs, including alert flags and section-level QC metrics. In the present study, sections with Median Genes per Spot < 300 were considered to have insufficient transcript complexity for robust downstream spatial analysis and were excluded from the core downstream analyses.

To enhance detection of viral transcripts, we constructed a composite reference genome comprising the human genome (hg38) and full-length HBV (GenBank: AB644283.1) and HCV (GenBank: AJ238799.1) genomes. Spatial gene expression data were processed with Space Ranger (10 × Genomics) using this composite reference (Fig. 1A), enabling simultaneous alignment of human and viral reads. Reads mapping to viral genomes were quantified on a per-spot basis. For downstream analysis, only uniquely mapped reads with mapping quality (MAPQ) ≥ 30 were retained. Multi-mapped and secondary alignments were excluded. Unique molecular identifiers (UMIs) were assigned to HBV/HCV gene features using custom gene annotations to summarize viral RNA abundance per spot. The Visium Spatial Gene Expression (fresh-frozen, v1) dataset used in this study was not analyzed as a strand-specific viral RNA assay; therefore, positive- and negative-strand HCV RNA could not be distinguished in the present dataset. Etiologic labels in this study reflect the clinical diagnosis assigned to each case; however, direct spatial viral-localization analyses were restricted to sections with detectable intrahepatic viral reads and were not inferred from clinical diagnosis alone.

### Serum viral load measurement

Serum viral loads for HBV and HCV were measured using quantitative real-time PCR (qPCR) with commercial reagent kits, according to the manufacturers’ protocols. Viral copy numbers were calculated using a standard-curve method and reported as international units per milliliter (IU/mL). These values were used to compare circulating viral burden with spatially resolved intrahepatic viral RNA signals.

### Serologic marker assessment

Serum HBV- and HCV-related serologic markers were obtained from clinical records. Institutional cut-off values were as follows: HBsAg, 1.0 IU/mL; HBeAg, 1.0 S/CO; anti-HBe, 50.0 C.O.I; and anti-HCV, 1.0 C.O.I. Values below these thresholds were interpreted as negative. For serum HBV DNA and HCV RNA assays, “Not detected” indicates values below the assay detection limit.

### Computational analysis

Raw sequencing data were first processed with the Space Ranger pipeline to generate spot-level gene expression matrices and aligned reads for the composite human + viral reference. Downstream analyses were performed in R. Using Seurat^13^, we conducted data normalization, scaling, dimensionality reduction (principal component analysis and Uniform Manifold Approximation and Projection, UMAP), clustering, and spatial mapping of gene expression patterns. Differentially expressed genes (DEGs) between anatomically or transcriptionally defined regions were identified and subjected to functional annotation using Gene Ontology (GO) enrichment and Reactome pathway analyses to interpret biological programs associated with each niche.

Cell–cell communication and ligand–receptor signaling networks were explored using SpatialCellChat/CellChat on spot-level spatial transcriptomic expression profiles, following the published workflow for spatial transcriptomics data^14^, Formal deconvolution was not performed prior to this analysis. Because Visium spots contain mixed-cell transcripts, the inferred ligand–receptor interactions were interpreted as exploratory spot-level or region-level signaling patterns rather than single-cell-resolved communication events. Viral genome coverage and read distributions across HBV and HCV genomes were visualized using Gviz^15^. To further interpret HCV-associated regions, we examined spot-level correlations between HCV-derived read abundance and hepatocyte-associated genes (*ALB, HNF4A, APOB, TTR*) and macrophage markers (*CD68, LYZ, MARCO, CD163*) and compared marker expression across Loupe-defined HCV_Negative, HCV_Near, and HCV_Positive regions.

### Figure generation and visualization software

Data-derived plots were generated by the authors using R version 4.4.3 (https://www.r-project.org/) together with relevant software packages according to the plot type. Spatial plots, including SpatialDimPlot, FeatureSpatialPlot, DimPlot, and PlotClusterTree, were generated using Seurat^13^ version 5.3.1 (https://satijalab.org/seurat/) and ggplot2 version 4.0.1 (https://ggplot2.tidyverse.org/). Heatmaps were generated using pheatmap version 1.0.13 (https://cran.r-project.org/package=pheatmap). Cell–cell communication network visualizations were generated using CellChat^14^ version 2.1.2 (https://github.com/jinworks/CellChat). Browser-based spatial views were generated using Loupe Browser version 9.0.0 (https://www.10xgenomics.com/support/software/loupe-browser/latest). Pathway-enrichment dot plots were generated in R using ggplot2 together with pathway annotations from the Reactome Pathway Database (https://reactome.org/). The schematic workflow in Fig. 1A was created by the authors using Microsoft PowerPoint for Mac version 16.107.3 (https://www.microsoft.com/microsoft-365/powerpoint).

### Statistics and reproducibility

All statistical analyses were performed in R. Unless otherwise specified, group comparisons were performed at the level of spatial capture spots or histology-defined regions within tissue sections using two-sided Wilcoxon rank-sum tests. Accordingly, reported p-values reflect spot-level or region-level comparisons and should not be interpreted as patient-population comparisons across etiologies. Because the core discovery analysis included one QC-passed case per etiology, these statistical results are presented as exploratory/descriptive rather than formal patient-level inference. Spatial clustering structures and viral-detection patterns were qualitatively reproducible across available cohorts, supporting the technical robustness of the workflow while not substituting for validation in larger cohorts.

## Supplementary Information

Supplementary Information 1.

Supplementary Information 2.

## Data Availability

Spatial transcriptome data (Visium) in this study has been deposited to DDBJ BioProject database under accession number DRR895143 and E-GEAD-1190. https://ddbj.nig.ac.jp/public/ddbj_database/dra/fastq/DRA025/DRA025923https://ddbj.nig.ac.jp/public/ddbj_database/gea/experiment/E-GEAD-1000/E-GEAD-1190/. https://ddbj.nig.ac.jp/public/ddbj_database/dra/fastq/DRA025/DRA025923. https://ddbj.nig.ac.jp/public/ddbj_database/gea/experiment/E-GEAD-1000/E-GEAD-1190/

## Code availability

All custom code used for viral-aware mapping and downstream spatial transcriptomic analyses are available from the corresponding author upon reasonable request. Unless otherwise specified in the Methods, data processing, downstream analyses, and visualizations were performed using standard functions and default settings of the indicated software packages. Custom code was used primarily for integrating the viral-aware mapping workflow, organizing outputs, and preparing analysis-ready inputs for downstream interpretation.
