# Supplementary material for: Spatial profiling of chronic liver disease: a pilot spatial case series
Source: Sci Rep. 2026 Apr 20;16:18223. doi: 10.1038/s41598-026-49400-7 (PMC13260902; doi:10.1038/s41598-026-49400-7)
Supplement: Supplementary file 2 — Supplementary Information 2. [file 41598_2026_49400_MOESM2_ESM.pdf]

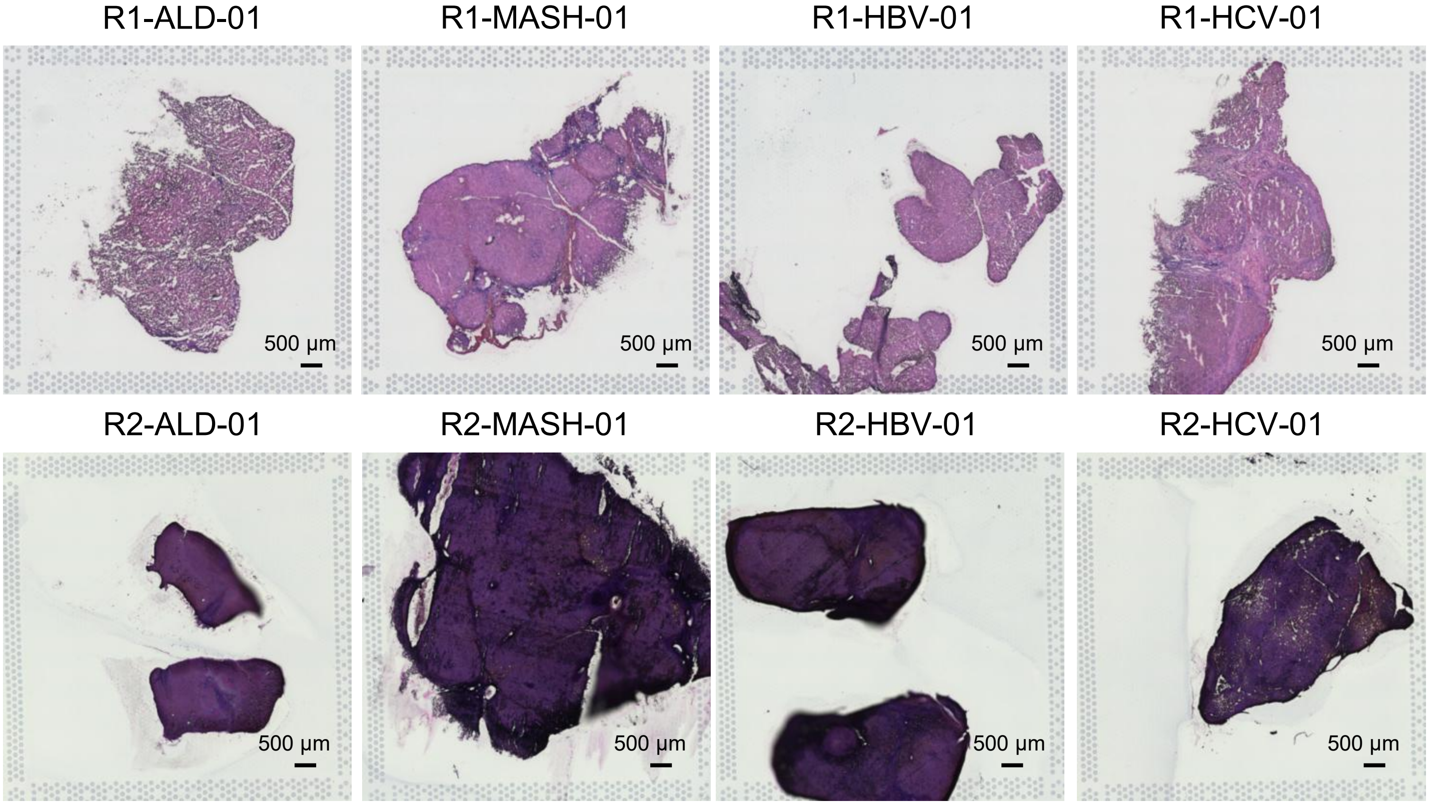

Supplementary Figure S1

Overview of all analyzed Visium sections. All eight analyzed sections (R1-ALD-01, R2-ALD-01, R1-MASH-01, R2-MASH-01, R1-HBV-01, R2-HBV-01, R1-HCV-01, and R2-HCV-01) are shown using a unified layout. For each specimen, the H&E/brightfield image with Visium spot coverage and the corresponding pathology-guided anatomical annotation are provided, together with sample ID, scale bar, and QC status. Seurat cluster maps and downstream module-based classifications are shown in the main figures and are therefore not repeated here.

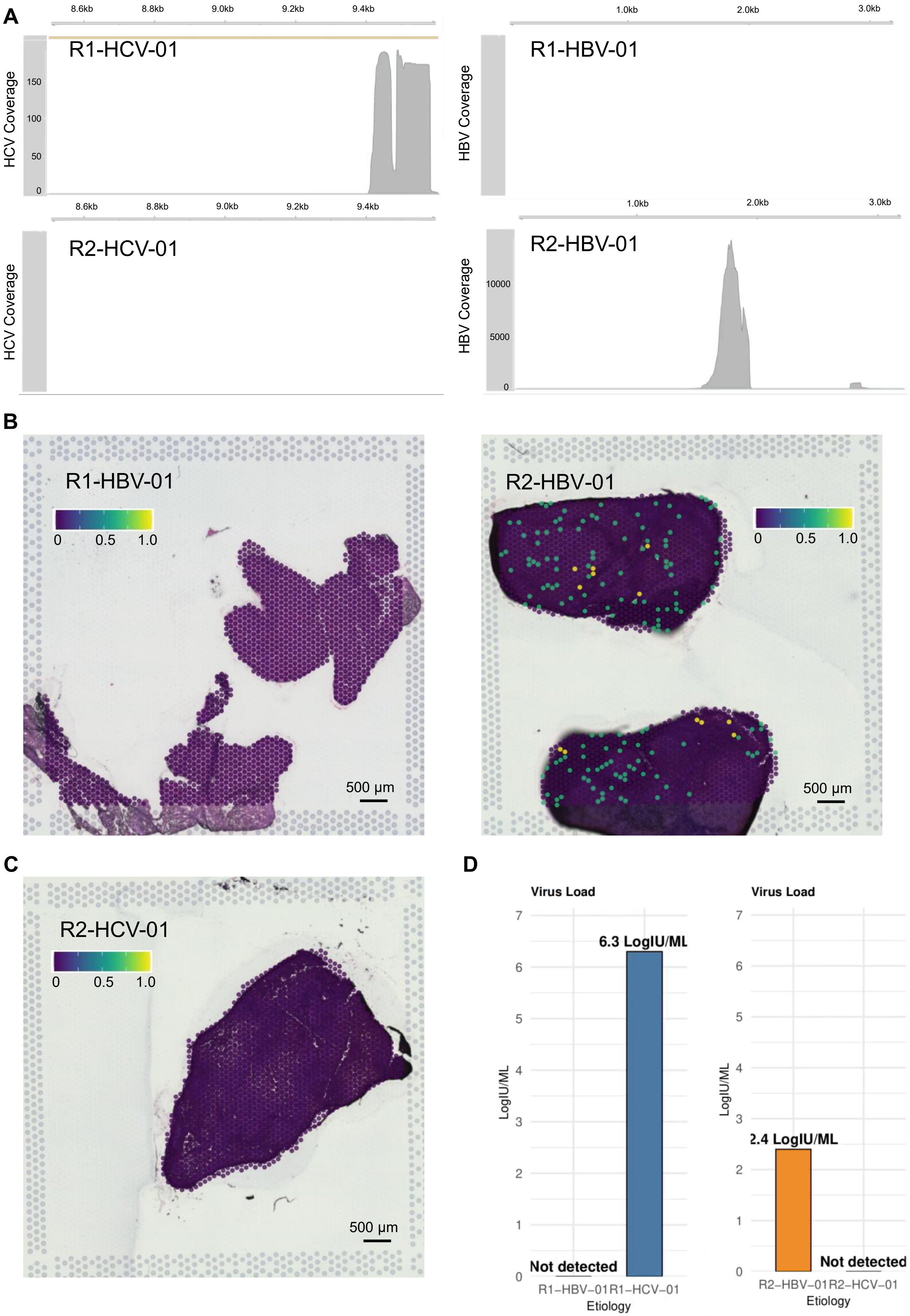
A
R1-HCV-01
HCV Coverage
8.6kb
8.8kb
9.0kb
9.2kb
9.4kb
150
100
50
0
R1-HBV-01
HBV Coverage
1.0kb
2.0kb
3.0kb
R2-HCV-01
HCV Coverage
R2-HBV-01
HBV Coverage
10000
5000
0
B
R1-HBV-01
0
0.5
1.0
500 µm
R2-HBV-01
C
R2-HCV-01
D
Virus Load
LogIU/ML
6.3 LogIU/ML
Not detected
R1−HBV−01
R1−HCV−01
Etiology
2.4 LogIU/ML
R2−HBV−01
R2−HCV−01

Figure S2

Supplementary Figure S2

Virus detection in liver tissues and corresponding serum virology.

(A) Reference coverage tracks for viral reads. HCV-derived reads were detected in R1-HCV-01, and HBV-derived reads were detected in R2-HBV-01.

(B) SpatialFeaturePlots of HBV-derived reads in the two HBV-profiled sections, R1-HBV-01 and R2-HBV-01. No detectable HBV-derived reads were observed in the analyzed R1-HBV-01 section, and the corresponding serum HBV viral load was below the detection limit. By contrast, R2-HBV-01 showed sparse HBV-derived reads, but this section did not pass QC for robust downstream analysis and was therefore not included in the core downstream analyses.

(C) SpatialFeaturePlot of HCV-derived reads in R2-HCV-01. No detectable HCV-derived reads were observed in this section, consistent with the corresponding serum HCV viral load being below the detection limit.

(D) Serum HBV DNA and HCV RNA levels measured by qPCR in the viral hepatitis cases from Run 1 and Run 2. In Run 1, R1-HCV-01 showed detectable HCV viremia (6.3 log IU/mL), whereas serum HBV viral load in R1-HBV-01 was below the detection limit. In Run 2, R2-HBV-01 showed detectable HBV viremia (2.4 log IU/mL), whereas serum HCV viral load in R2-HCV-01 was below the detection limit.

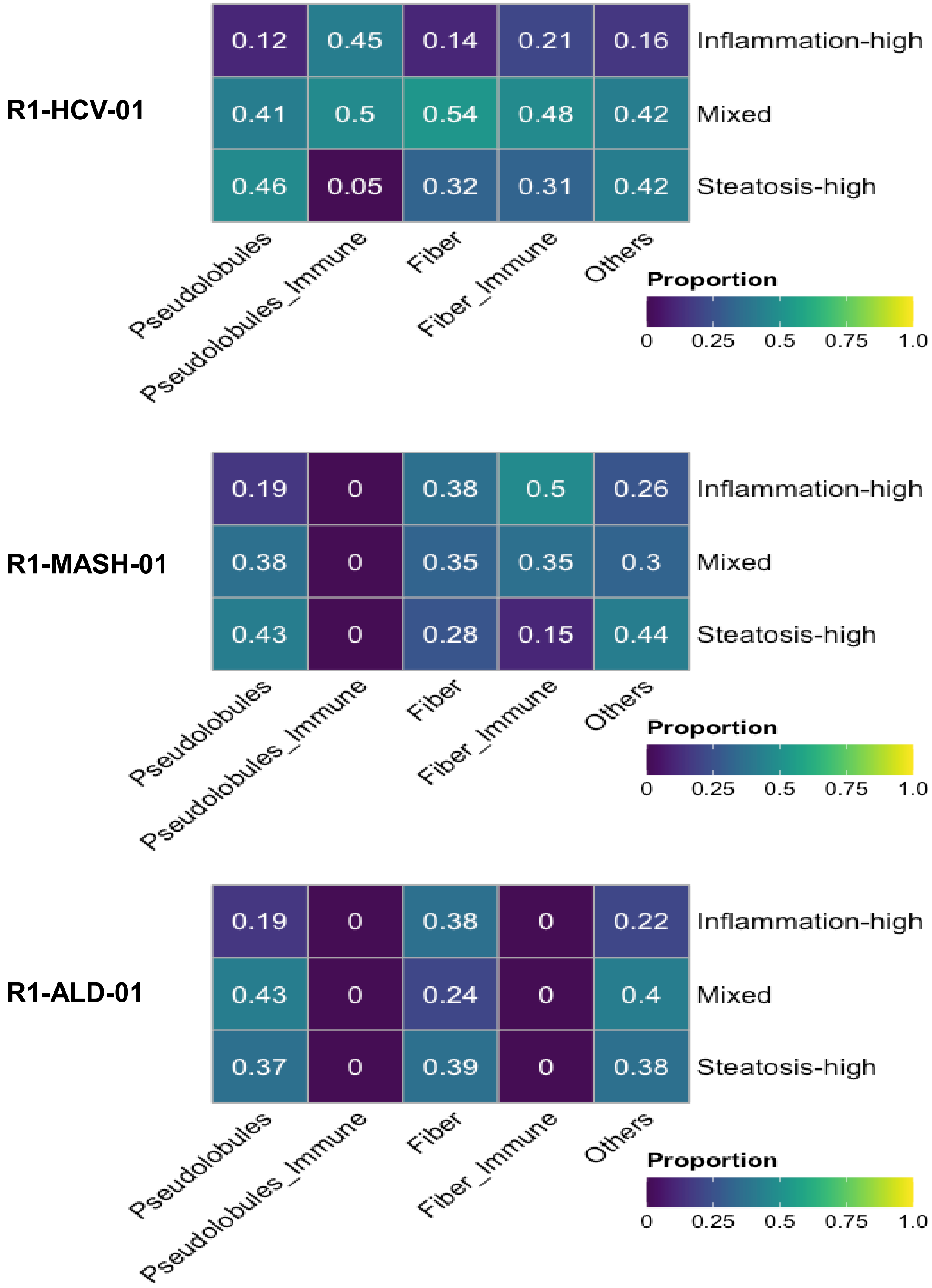

Supplementary Figure S3

Heatmap showing the proportions of Mixed, Steatosis-high, and Inflammation-high spots across broad histo-anatomical compartments in the ALD, MASH, and HCV discovery cases. Broad compartment labels were used to enable consistent cross-case comparison despite differences in the number of manually annotated subregions.

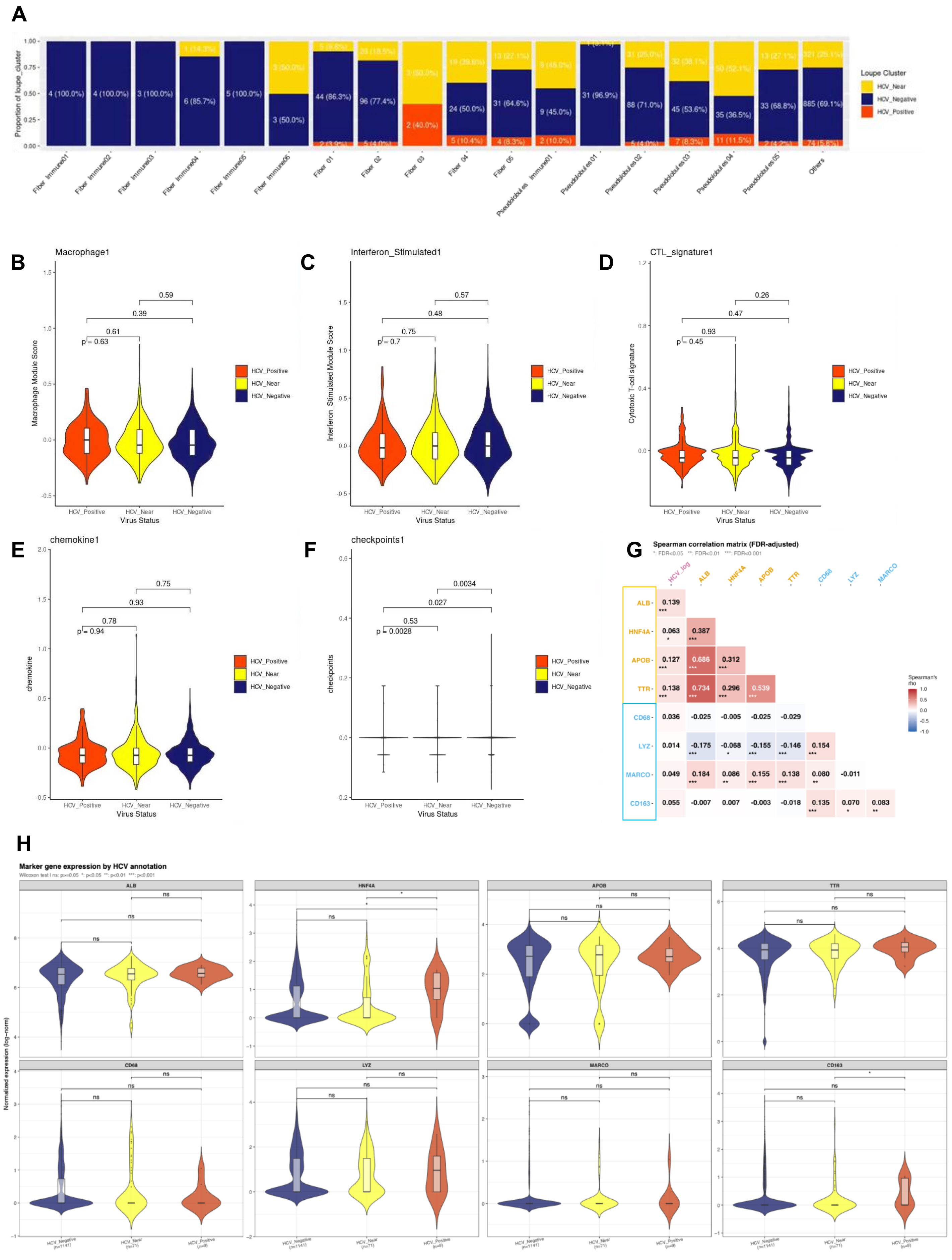
A
B
Macrophage1
C
Interferon_Stimulated1
D
CTL_signature1
E
chemokine1
F
checkpoints1
G
Spearman correlation matrix (FDR-adjusted)
H
Marker gene expression by HCV annotation
Loupe Cluster
HCV_Near
HCV_Negative
HCV_Positive
Virus Status

Figure S4

Supplementary Figure S4

Spatial immune gradient in HCV infection and immune-related gene expression. (A) Spatial distribution of cell type clusters across HCV_Positive, HCV_Near, and HCV_Negative regions, demonstrating a spatial immune gradient. Comparison of gene expression for (B) immune checkpoint molecules, (C) macrophage markers, (D) interferon-stimulated genes (ISGs), (E) T cell signatures, and (F) chemokines across different spatial regions. (G) FDR-adjusted Spearman correlation matrix between HCV-derived read abundance and hepatocyte/macrophage marker genes across all spots. (H) Expression of hepatocyte markers (ALB, HNF4A, APOB, TTR) and macrophage markers (CD68, LYZ, MARCO, CD163) across Loupe-defined HCV_Negative, HCV_Near, and HCV_Positive regions.

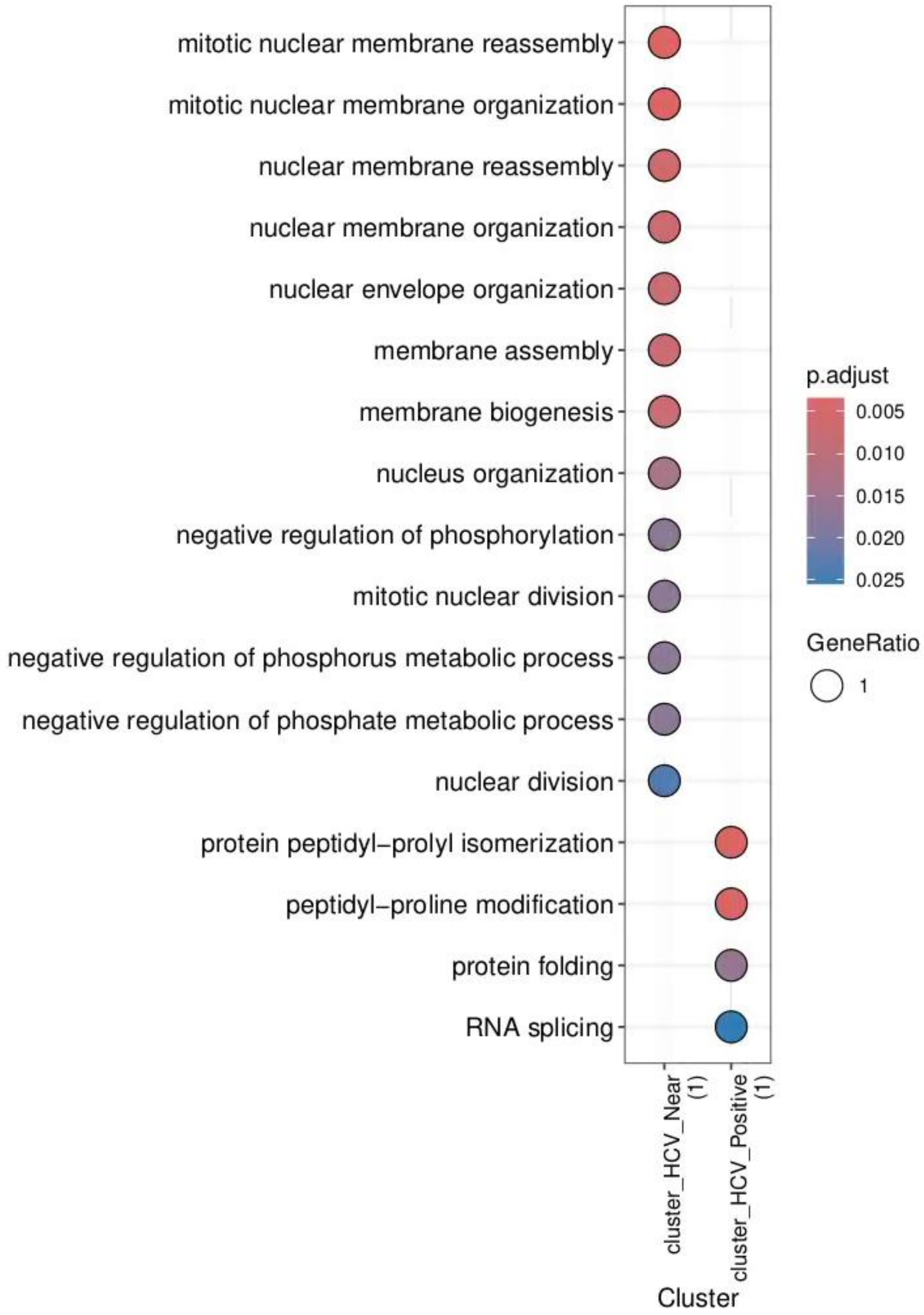

Supplementary Figure S5

Pathway analysis of HCV-proximal regions. Pathway enrichment analysis for spots located in HCV_Near regions, highlighting enrichment of mitotic control, RHO GTPase signaling, and mRNA splicing pathways.

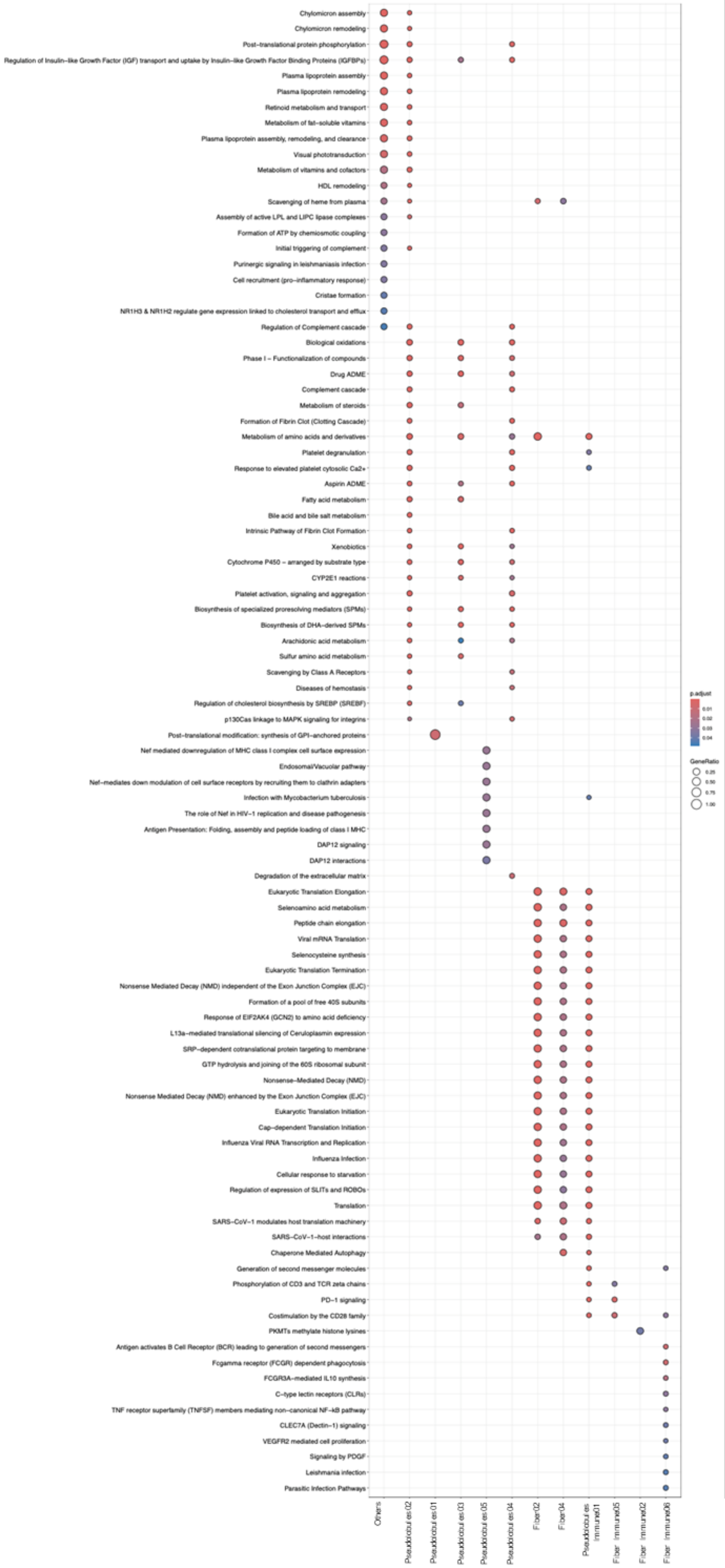

Supplementary Figure S6

Comprehensive pathway enrichment analysis.Comprehensive pathway enrichment analysis across all cell clusters, detailing a wide range of functional pathways.
